# Bile Acid Receptor Therapeutics Effects on Chronic Liver Diseases

**DOI:** 10.3389/fmed.2020.00015

**Published:** 2020-01-29

**Authors:** Vik Meadows, Lindsey Kennedy, Debjyoti Kundu, Gianfranco Alpini, Heather Francis

**Affiliations:** ^1^Richard L. Roudebush VA Medical Center, Indiana University School of Medicine, Indianapolis, IN, United States; ^2^Department of Biochemistry and Molecular Biology, Indiana University School of Medicine, Indianapolis, IN, United States; ^3^Division of Gastroenterology and Hepatology, Department of Medicine, Indiana University School of Medicine, Indianapolis, IN, United States

**Keywords:** bile acid, chronic liver disease, obeticholic acid, ursodeoxycholic acid, bile acid receptor

## Abstract

In the past ten years, our understanding of the importance of bile acids has expanded from fat absorption and glucose/lipid/energy homeostasis into potential therapeutic targets for amelioration of chronic cholestatic liver diseases. The discovery of important bile acid signaling mechanisms, as well as their role in metabolism, has increased the interest in bile acid/bile acid receptor research development. Bile acid levels and speciation are dysregulated during liver injury/damage resulting in cytotoxicity, inflammation, and fibrosis. An increasing focus to target bile acid receptors, responsible for bile acid synthesis and circulation, such as Farnesoid X receptor and apical sodium-dependent bile acid transporter to reduce bile acid synthesis have resulted in clinical trials for treatment of previously untreatable chronic liver diseases such as non-alcoholic steatohepatitis and primary sclerosing cholangitis. This review focuses on current bile acid receptor mediators and their effects on parenchymal and non-parenchymal cells. Attention will also be brought to the gut/liver axis during chronic liver damage and its treatment with bile acid receptor modulators. Overall, these studies lend evidence to the importance of bile acids and their receptors on liver disease establishment and progression.

## Introduction

Focal studies of hepatic secretion led to the critical analysis and understanding of bile and its circulation connecting the liver and intestine (1). Bile acids (BAs) are heterogenous compounds whose chemical and amphipathic properties result from the enzymatic breakdown of insoluble cholesterol (2). Since their isolation from bile, the field of BA chemistry has provided intensive study of their complex chemical nature and the physiological effects of their dynamic composition in circulating bile (2–5).

Hepatic BA build up leads to inflammation, necrosis, and apoptosis of various liver cells which then affects BA synthesis and transport perpetuating BA-induced damage (2, 3). Due to the extensive knowledge and research concerning hepatic BA formation and secretion, it is natural to assume BAs contribute to chronic liver damage. The increased synthesis of BAs in combination with interrupted BA signaling can lead to adverse effects in patients of chronic liver diseases. It is estimated that 1.5 billion people worldwide suffer from chronic liver diseases whose complications, cirrhosis and liver cancer, result in 2 million deaths globally (6). Chronic liver diseases have an increased burden in the health care system due to the frequent hospital readmissions, accompanying hepatic decompensation, and risk for infection as liver damage progresses (6). Many chronic liver diseases, such as primary sclerosing cholangitis (PSC) and non-alcoholic fatty liver disease (NAFLD), have minimal treatment options requiring liver transplantation as the only permanent remedy (6, 7).

Various therapeutics have been FDA-approved for clinical trials aiming to improve liver function and relieve adverse effects of disrupted BA signaling. This article serves as a brief review of BA signaling and function in various chronic liver diseases and their regulation of the gut microbiome.

## Circulation, Modification, and Function of Bile Acids

### Bile Acid Synthesis and Transport

**Figure 1:**
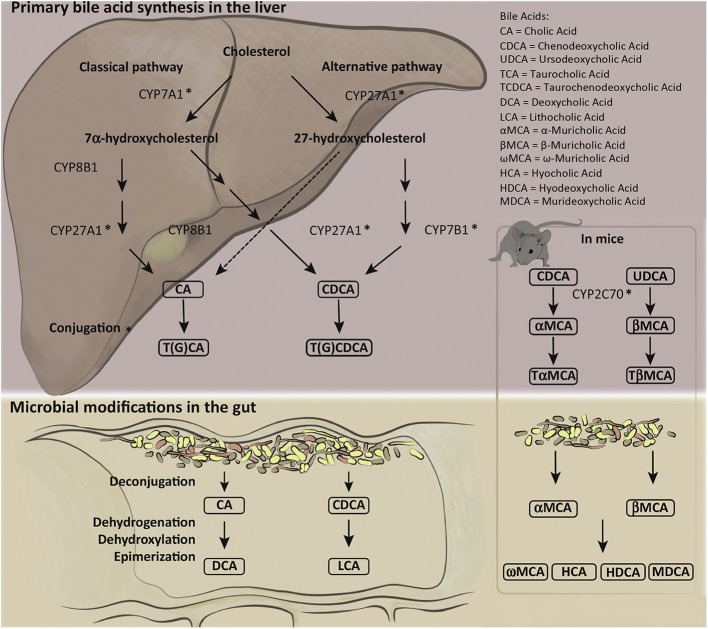
Human and mouse bile acid synthesis and conjugation. Cholesterol catabolism results in the creation of primary bile acids (BAs) through either the classical pathway, accomplished by Cytochrome P450 7A1 (CYP7A1), or the alternative/acidic pathway, conducted by Cytochrome P450 27A1 (CYP27A1). Alteration by various cytochrome P450 family of enzymes allows for the creation of cholic acid (CA) and chenodeoxycholic acid (CDCA). Primary BAs can become conjugated to glycine or taurine prior to secretion to the biliary ductules. Deconjugation and reconjugation occurs in the distal ileum through bacterial intervention creating secondary BAs: lithocholic acid (LCA) or deoxycholic acid (DCA). Mice have additional primary BAs: Ursodeoxycholic acid (UDCA) and α- and β-muricholic acid. The murine specific primary BAs created by cytochrome P450 2C70 (CYP2C70) can also be conjugated to glycine or taurine prior to secretion into bile duct and alteration by gut microbiota into secondary BAs. Figure reprinted with permission from Molinaro et al. Trends Endocrinol Metab.

The catabolism of cholesterol into BAs in the parenchyma is tightly regulated by over 17 enzymes, preferentially expressed in the liver, that are involved in the synthesis and alteration of BAs into bile salts (8, 9). Impairment of these mechanisms can result in cholestasis, liver damage, impaired lipid metabolism, and other maladies (3, 5, 8). Modifications and conjugations of BAs affect their solubility, hydrophobicity, and receptor binding affinity (8, 10). BA synthesis and conjugations are summarized in Figure 1 (reprinted with permission from Molinaro et al. Trends Endocrinol Metab).

Bile acids are secreted into the bile canaliculi by hepatocytes, draining to the bile ducts located in the portal triad. Cholangiocytes are the epithelial cells lining the bile ducts and assist in BA modification and circulation by cholehepatic shunting, the process in which BAs are reabsorbed from the bile and returned to hepatocytes (11–14). The intrahepatic bile duct system allows BAs to flow into the intestinal lumen through the common hepatic duct in response to food ingestion to assist in emulsification, metabolism and absorption of dietary lipids and fat-soluble vitamins (A, D, E, and K). Alternatively, BAs are deposited in the gallbladder for storage and prevention of cholesterol-crystallization and gallstone formation (10, 15). Approximately 95% of BAs are reabsorbed in the distal ileum by the apical sodium-dependent bile acid transporter (ASBT; alternatively known as ileal bile acid transporter, IBAT) and delivered to the liver through the portal system via enterohepatic or portal circulation (16–18). The gut microbiome is capable of deconjugating primary bile acids and converting them to secondary bile acids prior to absorption or fecal excretion which affects gut microbiome community, BA pool, and liver health (8, 19, 20).

Aside from their important roles in digestion, BAs can behave as signaling molecules in carbohydrate and lipid metabolism, energy expenditure, and hepatic disease (20–22). BAs and activation of their downstream targets including G-protein-coupled bile acid receptor (TGR5), transforming growth factor-α (TGF-α) and sphingosine-1-phosphate receptor-2 (S1PR2) stimulate cholangiocyte proliferation and contribute to the progression of cholangiocarcinoma [CCA, *in vivo* and *in vitro* (21–25)]. Alternatively, Farnesoid X Receptor (FXR) is down regulated in hepatocellular carcinoma (HCC) (26). It has been shown that FXR via increased CYP450 epoxygenase activity suppress NF-κB signaling thereby reducing hepatic inflammation (27, 28). Further exploration into the anti-inflammatory role of FXR and assessment of BA direct or indirect targets may provide understanding of chronic cholestatic disease establishment and progression.

### Intrahepatic and Extrahepatic Bile Acid Modification

The catabolism of cholesterol results in the formation of the primary BAs, cholic acid (CA) or chenodeoxycholic acid (CDCA), through the major (classical) pathway or the minor (alternative/acidic) pathway, respectively (29). Cholehepatic shunting alters the BA pool via biliary ASBT transport, multidrug resistance cassette 3 (MDR3, human; multidrug resistance cassette 2, mice), and organic solute transporter α-β (OSTα-β) BA secretion into the peribiliary plexus prior to reaching the hepatic sinusoids (30). Ileal bile acid binding protein (IBABP) is expressed in large cholangiocytes to sequester BAs preventing biliary cytotoxicity (30, 31). CA and CDCA/Ursodeoxycholic acid (UDCA) are converted to deoxycholic acid (DCA) and lithocholic acid (LCA), respectively, via 7α/β-dihydroxylation by various species of the commensal gut microbiota in the gastrointestinal tract (32). Human secondary BAs (DCA and LCA) are capable of being circulated back to the liver via enterohepatic circulation leading to an increased hepatic levels of damaging hydrophobic BAs (32).

## Dysregulation of Bile Acids in Chronic Liver Diseases

### PSC and PBC

PSC and Primary Biliary Cholangitis (PBC) are rare cholestatic liver diseases that affect the biliary system. PSC is an idiopathic disease with cholestasis, inflammation and eventual fibrosis resulting from strictures of intra- and extrahepatic bile ducts (33). PSC is one of the most common causes for liver transplantation (LT) (33). Due to its heterogenous and spontaneous progression, effective medical therapies have not yet been developed (33). Fat-soluble vitamin deficiency can occur in PSC patients as a result of decreased bile flow and secretion. It has also been shown that PSC has a positive correlation with ulcerative colitis (UC), a form of inflammatory bowel disease (IBD). PSC/IBD patients display altered BA fecal excretion and decreased gut microbiome diversity compared to healthy or IBD patient controls (34). Patients with PSC have decreased expression of hepatic FXR, TGR5, and S1PR2 (35). The multidrug resistance cassette 2 knock-out mouse (MDR2^−/−^) is a mouse model utilized to mimic the PSC phenotype including increased cholestasis, intrahepatic bile duct mass and hepatic inflammation due to hepatic BA build up (36, 37). This murine model has been useful for identifying effects of potential therapeutics, such as UDCA. Meng et al. reported that UDCA treatment in Mdr2^−/−^ mice reduced serum TBA, elevated hepatic expression of BA transporters, and reduced hepatic inflammation and collagen deposition (36).

PBC is a chronic auto-immune disease, predominantly affecting middle-aged women, that results in biliary ductopenia and cholestasis. Li et al. reported elevated serum levels of total BAs (TBA) and FGF19 in cirrhotic PBC patients compared to healthy controls and non-cirrhotic PBC patients (38). Similarly, Trottier et al. demonstrated elevated BAs in serum samples from both PBC and PSC compared to healthy controls (39). Ursodeoxycholic acid (UDCA), an epimer of CDCA, was the first FDA-approved treatment for PBC. Despite increased bile flow, lower liver enzyme levels, and decreased serum BA levels, one in three PBC patients will have a limited or no response to treatment, strengthening the need for effective therapeutic intervention of PBC progression (33, 40–45).

### NAFLD

NAFL and non-alcoholic steatohepatitis (NASH) are two of the most common hepatic diseases worldwide due to an increase of sedentary lifestyle and consumption of a high-fat/high-cholesterol diet (46, 47). Its prevalence has demonstrated positive correlation with an increasing number of obese and type II diabetic patients (46). Currently, there are no approved therapies for the treatment of NAFL and NASH aside from a change of diet and exercise for gradual weight loss.

BA signaling is disrupted in NAFL and NASH patients yielding great interest in the search for exogenous methods of BA regulation (48, 49). Mouzaki et al. uncovered greater fecal BA secretion and increased primary to secondary BA ratio in NASH patients compared to healthy controls (49). Ferslew et al. found elevated serum BAs in NASH patients, compared to healthy controls, with an increase of taurine- and glycine-conjugated BAs (48). Benedict and Zhang proposed that FXR suppression of hepatic inflammation may ameliorate NAFLD progression (50).

### HCC and CCA

HCC is currently the third leading cause of cancer deaths worldwide. HCC affects parenchymal cells in the liver, which make up to 70% of the liver tissue. HCC develops in chronic liver disease or cirrhotic liver patients and is usually detected through various imaging methods prior to diagnosis. Patients with HCC can be asymptomatic or present with a range of symptoms including cirrhosis-related pain. The underlying chronic liver injury, and difficulty in diagnosis, both contribute to HCCs high mortality. CCA is a rare but devastating cancer with poor prognosis. Patients present with jaundice, pruritus (intense itch) and acholic (pale) stool due to reduced bile and bilirubin excretion. Due to the intimate relationship between hepatocytes, cholangiocytes and BAs it is important to investigate BA signaling in the establishment and progression of HCC and CCA.

Demonstrating a shift in the BA pool during cancer development, Changbumrung et al. reported elevated ratios for trihydroxy to dihydroxy BAs and for glycine-conjugated to taurine-conjugated BAs in patients with HCC and CCA compared to healthy patients (51). Luo et al. demonstrated a similar trend with elevated glycine-conjugated BAs in hepatic injury patients, ranging from hepatitis B viral infection to cirrhosis, compared to healthy controls suggesting taurine-conjugated BAs as a potential sensitive biomarker for liver injury (52). This increase in BA pool size is due to reduced inhibition of BA synthesis. FXR activation has been indicated to have anti-cancer properties, with decreased expression in progressing human HCC lesions, since its downstream effects include inhibition of BA synthesis and cell proliferation (26, 53). Guo et al. reported decreased FXR expression in HCC tumor lesions, indicating a hindering role in HCC development and progression (26). Wolfe et al. found a decrease of FXR expression in HCC tumor lesions, compared to normal liver tissue, with increasing tumor development stage (53). Similarly, Liu et al. reported decreased small heterodimer partner (SHP) and FXR expression in human HCC compared to paired healthy control (54). Erice et al. demonstrated FXR expression in CCA samples negatively correlates with advanced cancer progression and lymph node invasion. In contrast to FXR, TGR5 expression is elevated in CCA tumors compared to controls (25). This discovery is likely due to cholangiocyte requirement of TGR5 for BA-induced proliferation and anti-apoptosis signaling (55, 56). Additional BA receptors, such as S1PR2, have been found to have elevated expression in human CCA tumors, as shown by Liu et al. (57). Taken together these studies indicate that altered BAs may serve to enhance HCC and CCA progression during disease development.

## Current Bile Acid-Related Therapies of Chronic Liver Diseases

### Bile Acid Therapies

The use of synthetic or naturally occurring BAs to reduce gallstone formation, aid in lipid absorption following gastric surgeries, and assist in reduction of cholestasis has increased in recent years. UDCA is a hydrophilic BA conjugated to undergo enterohepatic circulation or deconjugated and converted into LCA by the gut microbiome and excreted into feces. UDCA was the first FDA-approved therapy for PBC patients exhibiting altered serum liver enzyme levels (41). UDCA has been a staple therapy due to its ability to prevent gallstone formation, increasing bicarbonate secretion to prevent acidification of bile, and positive side effect profile as compared to treatment with CDCA (18, 41, 58). In many PBC patients, long-term UDCA treatment when administered at early stage of disease increases survival rate, lowers liver enzyme serum levels, and improves liver histology (41, 43, 59, 60). Regardless of its beneficial effects, 30–40% of PBC patients do not respond to UDCA treatment.

UDCA treatment can improve liver histology and serum ALT/ASP levels in PSC patients, however, data supporting any long-term efficacy or long-term survival are lacking (61). Moreover, UDCA prescribed at high doses increased medical complications and mortality in PSC patients (61–64). PSC patients treated with norUDCA, a side chain shortened homolog of UDCA, exhibited reduced ALP serum levels compared to placebo in a dose-dependent manner, however norUDCA's effects on PSC progression, long-term survival, and mortality have not been investigated (65).

### Bile Acid Receptor/Transporter Agonists and Antagonists

Obeticholic acid (OCA, Ocaliva) is an FDA-approved, synthetic derivative of CDCA and is a high affinity ligand for the nuclear bile acid receptor, FXR (40). FXR is a nuclear BA receptor that when expressed is capable of reducing BA synthesis, increasing expression of BA transporters and modulating lipoprotein metabolism (66). OCA has been studied as a potential therapeutic drug in the treatment of various chronic liver diseases (40, 44). Following Phase II and Phase III clinical trials, it has been suggested that OCA treatment can be delivered in conjunction with UDCA in PBC, or as a monotherapy in patients who do not tolerate UDCA, respectively (67, 68). Adverse events including pruritus occurred in nearly all patients and was observed to occur in a dose dependent manner (67, 68). The beneficial effects of OCA as a monotherapy are currently being investigated in various clinical trials (7, 67, 69). The use of OCA has promising effects on improving liver enzyme levels, lowering plasma bilirubin and IgM levels, and has recently been shown to improve or stabilize liver fibrosis and biliary injury in PBC patients with cirrhosis (68, 69). Despite promising outcomes in PBC patients, long-term effects of OCA on disease progression and patient survival are still being investigated.

In the Farnesoid X Receptor Ligand Obeticholic Acid in NASH Treatment (FLINT) clinical trial, NASH patients treated with OCA presented with improved fibrosis scores, elevated low-density lipoprotein (LDL) and increased average weight loss compared to placebo control (46, 70). NASH patients treated with OCA also had increased ALP levels compared to placebo control (46). The effects of OCA on insulin resistance and the observed improvement of lipid absorption were not sustained following termination of OCA treatment (46). Further investigation into the short-term and long-term effects of OCA on liver function and injury are warranted and are actively being explored in the Randomized Global Phase III Study to Evaluate the Impact on NASH with Fibrosis of Obeticholic Acid Treatment (REGENERATE) clinical trial assessing FXR activation in the treatment of NASH (71). Translational Research and Evolving Alcoholic hepatitis Treatment (TREAT) consortium conducted phase II clinical trial utilizing OCA on patients with moderately severe alcoholic hepatitis (AH) that was completed in 2018, however currently no data is available on this study (72). The Phase 3 Study of Obeticholic Acid in Patients with Primary Biliary Cirrhosis (POISE) double-blind, placebo controlled clinical trial has been one of the few to publish results demonstrating OCA's long-term safety and effectiveness (69). POISE study data showed that three-year OCA treatment reduced or stabilized hepatic collagen deposition and ductular injury in PBC patients with cirrhosis (69). The Combination OCA and Statins for Monitoring of Lipids (CONTROL) clinical trial study found increased LDL in NASH patients treated with OCA (5, 10, or 25 mg) indicating altered lipid metabolism (73). The observed increase in LDL cholesterol was reduced in NASH patients concurrently treated with OCA and atorvastatin, a statin utilized to reduce endogenous cholesterol production (73). The authors noted that these two drugs were well-tolerated when utilized together, addressing the observed increase in LDL cholesterol following OCA treatment in NASH patients, but remained inconclusive with respect to the combinatorial effect on hepatic injury (70, 73). Eaton et al. observed that decompensated patients with cirrhotic PSC and PBC, OCA treatment led to the development of jaundice and elevated liver enzyme levels (74). While the long-term benefits of OCA are still being evaluated in various liver diseases, it is still being explored as a potential.

During normal enterohepatic circulation, IBAT/ASBT is responsible for the reabsorption of BAs in the intestinal tract prior to secretion into the portal blood system (75). Additionally, ASBT is responsible for cholehepatic shunting of BAs between cholangiocytes and hepatocytes, which ultimately increases hepatic BA pool. A common symptom of chronic cholestatic liver disease is pruritus, intense itching of the dermis due to increased BA deposition (75). IBAT/ASBT inhibitors are potential therapeutic candidates for this detrimental symptom (76). Pilot studies conducted with various synthetic IBAT/ASBT inhibitors have demonstrated variable results. The Al-Dury et al. study reported patients who continued through A4250 (synthetic IBAT/ASBT inhibitor) received relief from itching, but pruritus returned during wash-out and BA sequestrant treatment. Despite improvement of pruritus and lowered serum BAs, many patients in the A4250 study dropped out early due to abdominal pain and diarrhea (76). Similar benefits were identified in the pilot study of GSK2330672 (synthetic IBAT/ASBT inhibitor), PBC patients resulted with lowered serum BAs and increased serum FGF19. The most common adverse events in this treatment cohort were headaches and diarrhea, which provides reason to limit the treatment length in patients with pruritus (77).

## BA Receptor Agonist Effects on the Gut/Liver Axis During Liver Disease

BA species affect and regulate gut microbial species composition (32). BA species and concentrations in different portions of the gastrointestinal tract can result in increased side effects including increased intestinal permeability and BA-induced diarrhea (78). Elevated hydrophobic BAs in the colon are capable of inducing inflammation which is reduced following CDCA treatment alleviating the increased toxicity from insoluble BA concentrations and mast cell secretory factors (i.e., histamine or nerve growth factor NGF) (78, 79).

A small cohort of PBC patients exhibited altered gut microbiome composition, compared to healthy controls, which was partially reversed following 6-month UDCA treatment (80). This study found eight-PBC associated genera and a reduction in normal gut microbiome associated microbial members including *Faecalibacterium, Bacteroides, Sutterella*, and *Oscillospira* spp. A study from Selmi et al. implicated increased *N. aromaticivorans* population is responsible for the induction of PBC (81). Tang et al. reported increased epithelial infiltration of *Enterobacteriaceae* in PBC patients indicating increased permeability and decreased gastrointestinal immune response in diseased patients (80).

The gastrointestinal epithelial barrier plays an important role in maintaining BA enterohepatic circulation homeostasis and reduced inflammation from lipopolysaccharide (LPS) leakage. BA circulation is necessary to avoid damaging increases in colonic BAs that result in inflammation and intestinal damage (78). Song et al. found that CDCA was capable of reducing paracellular permeability and increased cell-to-cell tight junctions via FXR activation in mice (82). Removal of commensal gut microbiota in Mdr2^−/−^ mice resulted in increased serum liver enzyme levels, cholangiocyte senescence, and circulating primary BAs (37). Alternatively, Li et al. discovered a depletion of BA induced damage to the colon and reduced mast cell activation and degranulation in FXR^−/−^ mice or *Z*-guggulsterone treated mast cells (79).

The role of the gut microbiome in BA homeostasis is still being investigated. The disruption of the gut-liver axis can allow infiltration of microbes, or their metabolic products, into the enterohepatic circulation to prolong disease (83). Chronic liver disease patients all maintain their own gut microbiome signature that inherently play a role in disease development. Future studies of BA-associated therapeutics should consider effects on the gut microbiome and their metabolites in relation to changing BA pool/circulation and chronic liver disease.

## Conclusion

**Figure 2:**
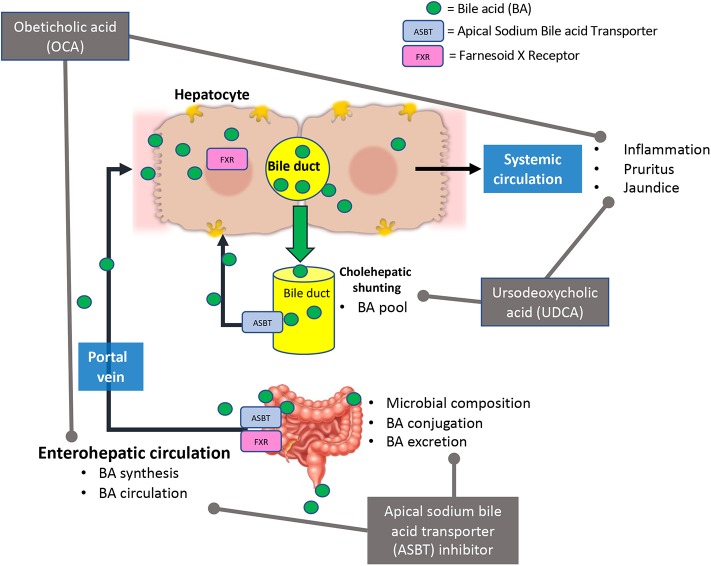
Current bile acid receptor therapeutics and their effects in bile acid signaling. Briefly, Obeticholic acid (OCA) reduces bile acid (BA) synthesis, circulating BA levels, and hepatic inflammation. This treatment may cause adverse effects such as pruritus and jaundice (in decompensated PBC patients). Ursodeoxycholic acid (UDCA) alters BA pool and reduced hepatic inflammation and damage. In some cases, UDCA treatment has led to development of pruritus. Apical sodium bile acid transporter (ASBT) inhibitors affect bile acid synthesis, circulation, secretion, and microbial composition in the intestine.

BA signaling, and therapeutics that alter them, have effects on the gut microbiome and organs outside of the liver. The use of BA receptor agonists and antagonists and their indirect effects (BA pool, synthesis, circulation, and the gut microbiome composition) is summarized in Figure 2. The human and mouse gut microbiome fluctuates with the increase or decrease of BAs, which can lead to increased inflammation and intestinal malabsorption (19, 37, 80, 84–86). In depth exploration of BA signaling and circulation during chronic liver diseases may provide insight to disease amelioration or treatment strategies. Understanding the role of the gut microbiome on BA modification and circulation may allow for innovative concurrent treatment for the reductions of inopportune side effects of the currently investigated treatments, OCA and UDCA.

## Author Contributions

VM and HF drafted manuscript. VM, LK, DK, GA, and HF edited and approved final version of manuscript. VM and HF arranged figures.

### Conflict of Interest

The authors declare that the research was conducted in the absence of any commercial or financial relationships that could be construed as a potential conflict of interest.

## Abbreviations

ALKP/ALP: Alkaline phosphatase
ASBT: apical sodium bile acid transporter
AST: aspartate aminotransferase
Bas: bile acids
FXR: Farnesoid X receptor
CA: cholic acid
CCA: cholangiocarcinoma
CDCA: chenodeoxycholic acid
DCA: deoxycholic acid
HCC: hepatocellular carcinoma
IBABP: ileal bile acid binding protein
IBD: irritable bowel disease
IBAT: ileal bile acid transporter
LCA: lithocholic acid
LDL: low density lipoprotein
MDR2: multidrug resistance cassette 2
MDR3: multidrug resistance cassette 3
NAFLD: non-alcoholic fatty liver disease
NASH: non-alcoholic steatohepatitis
OCA: Obeticholic acid
OSTα-β: organic solute transporter α-β
PBC: primary biliary cholangitis
PSC: primary sclerosing cholangitis
TBA: total bile acid
TGR5: Takeda G protein coupled receptor 5
UDCA: Ursodeoxycholic acid.
